# The complete mitochondrial genome of *Matuta victor* (Decapoda: Matutidae) from Beibu Bay

**DOI:** 10.1080/23802359.2020.1861999

**Published:** 2021-01-20

**Authors:** Lianghua Huang, Shengping Zhong, Yonghong Liu, Guoqiang Huang, Xiuli Chen

**Affiliations:** a Institute of Marine Drugs, Guangxi University of Chinese Medicine, Nanning, China; b Guangxi Engineering Technology Research Center for Marine Aquaculture, Guangxi Institute of Oceanology Co., Ltd, Beihai, China; c Guangxi Key Laboratory of Aquatic Genetic Breeding and Healthy Aquaculture, Guangxi Academy of Fishery Sciences, Nanning, China

**Keywords:** Mitochondrial genome, *Matuta victos*, Decapod

## Abstract

The true crabs (Brachyura) including Calappidea are one of the most diverse groups of Decapod crustaceans However, despite their great diversity and commercial importance, phylogenetic and classification relationships within Calappidea are still complicated and controversial. In this study, we report the first complete mitochondrial genome of *Matuta victos*. The mitogenome has 17,782 base pairs (70.1% A + T content) and is made up of a total of 37 genes (13 protein-coding, 22 transfer RNAs and two ribosomal RNAs), plus a putative control region. This study will provide useful molecular resources for clarifying evolutionary and phylogenetic confusion within Calappidea.

The Brachyura (Crustacea: Decapoda), known as the true crabs, is the most species-rich clade of Decapod Crustacea containing more than 7250 valid species around the world (Basso et al. 2017), many of which are economically and morphologically diverse group such as the box crabs (Brachyura: Calappidea). Early taxonomy of the Brachyura was largely morphology-based phylogenetic analyses. However, due to their high morphological diversity and extreme ecological diversity, the phylogenetic relationships among the brachyuran families are poorly understood and controversial (Tsang et al. 2014). The Calappidea appeared relatively early in the evolution of Brachyura, however, until now the taxonomic position of Calappidea has not been determined yet (Zhong et al. 2018; Lu et al. 2020). Complete mitochondrial genomes are becoming increasingly commonly used in reconstructing phylogenetic relationship and evolutionary classification. Unfortunately, up to now, the mitogenomic sequences of the box crabs are still limited. Here, we report the first complete mitochondrial genome of *Matuta victor*, which will provide a useful genetic resource for and phylogenetic analyses among Brachyura.

Tissue samples of *M. victos* from five individuals were collected from Guangxi province, China (Beihai, 21.057591 N, 109.085712 E) using local crab trap, together with the whole body specimen (#GQ0158) were deposited at Marine biological Herbarium, Guangxi Institute of Oceanology, Beihai, China. The total genomic DNA was extracted from the muscle of the specimens using an SQ Tissue DNA Kit (OMEGA, Guangzhou, China) following the manufacturer’s protocol. DNA libraries (350 bp insert) were constructed with the TruSeq NanoTM kit (Illumina, San Diego, CA) and were sequenced (2 × 150bp paired-end) using the HiSeq platform at BGI Company, China. Mitogenome assembly was performed by MITObim (Hahn et al. 2013). The complete mitochondrial genome of *M. planipes* (GenBank accession number: NC_039351) was chosen as the initial reference sequence for MITObim assembly. Gene annotation was performed by MITOS (http://mitos2.bioinf.uni-leipzig.de).

The complete mitogenome of *M. victos* (GenBank accession number: MT416712) is slightly bigger than *M. planipe* in size. The total length is 15,782 bp and contains a typical set of 13 protein-coding genes (PCGs), 22 transfer RNA genes, two ribosomal RNA genes, and a putative control region. The overall base composition of the mitogenome is estimated to be A 34.8%, T 35.3%, C 18.8% and G 11.1%, with a high A + T content of 70.1%, which is similar, but a slightly lower than *M. planipe* among genus *Matuta* (70.8%) (Lin et al. 2018). The concatenated nucleotides sequences of 13 PCGs were used to reconstruct phylogenetic relationship with the maximum-likelihood method using PhyML 3.0 Web Server (http://www.atgc-montpellier.fr/phyml/). The result suggests that *M. victos* and *M. planipe* had the most close relationship and formed a Calappoidea group with *Calappa bilineata* (Figure 1), which is consistent with the phylogenetic position of Calappoidea within Brachyura reconstructing by comparative mitogenomic analyses (Lu et al. 2020). The complete mitochondrial genome sequence of *M. victos* will be useful for better understanding the evolutionary and phylogenetic classification in Calappoidea.

**Figure 1.**
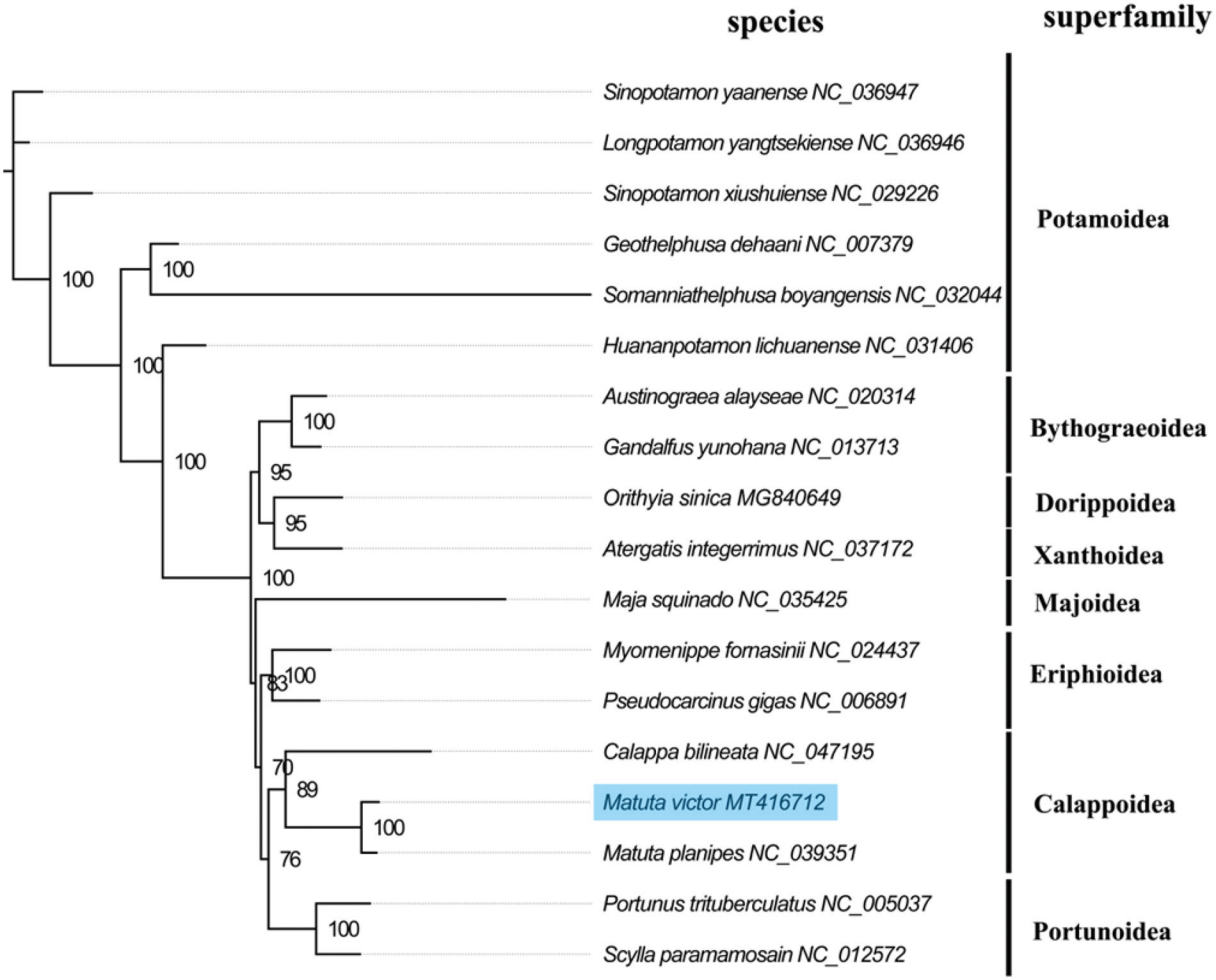
Phylogenetic tree of 18 species in Brachyuran. The complete mitogenomes is downloaded from GenBank and the phylogenic tree is constructed by the maximum-likelihood method with 100 bootstrap replicates. The bootstrap values were labeled at each branch nodes.

## Data Availability

The data that support the findings of this study are openly available in [National Center for Biotechnology Information] at [https://www.ncbi.nlm.nih.gov/nuccore], reference number [MT416712]. The raw data have been deposited into CNGB Sequence Archive (CNSA) of China National GeneBank DataBase (CNGBdb) with accession number CNP0001357.
